# Pollen chemical and mechanical defences restrict host-plant use by bees

**DOI:** 10.1098/rspb.2023.2298

**Published:** 2024-03-13

**Authors:** Sébastien Rivest, Madhupreeta Muralidhar, Jessica R. K. Forrest

**Affiliations:** ^1^ Department of Biology, University of Ottawa, Ottawa, ON, Canada K1N 6N5; ^2^ Rocky Mountain Biological Laboratory, Crested Butte, CO 81224, USA

**Keywords:** pollen, bees, secondary metabolites, defence compounds, pollinators, flowers

## Abstract

Plants produce an array of chemical and mechanical defences that provide protection against many herbivores and pathogens. Putatively defensive compounds and structures can even occur in floral rewards: for example, the pollen of some plant taxa contains toxic compounds or possesses conspicuous spines. Yet little is known about whether pollen defences restrict host-plant use by bees. In other words, do bees, like other insect herbivores, tolerate the defences of their specific host plants while being harmed by non-host defences? To answer this question, we compared the effects of a chemical defence from *Lupinus* (Fabaceae) pollen and a putative mechanical defence (pollen spines) from Asteraceae pollen on larval survival of nine bee species in the tribe Osmiini (Megachilidae) varying in their pollen-host use. We found that both types of pollen defences reduce larval survival rate in some bee species. These detrimental effects were, however, mediated by host-plant associations, with bees being more tolerant of the pollen defences of their hosts, relative to the defences of plant taxa exploited by other species. This pattern strongly suggests that bees are adapted to the pollen defences of their hosts, and that host-plant use by bees is constrained by their ability to tolerate such defences.

## 1. Introduction

Plants have developed an array of chemical and mechanical defences in response to selection imposed by herbivores and pathogens [1–3]. While these defences are often associated with vegetative tissues and mediate antagonistic interactions, nectar and pollen—the most common floral rewards—also frequently contain toxic secondary metabolites [4–7], and many types of pollen exhibit conspicuous spines [8–10]. The concentration of secondary metabolites in pollen can even match that of vegetative tissues [6]. Therefore, not only herbivores in the classical sense (i.e. consumers of vegetative tissue), but also potentially pollinators, must confront plant defences. In insect herbivores, host-plant use is strongly restricted by plant defences, as herbivores often possess physiological adaptations allowing them to overcome some host defences, while they are harmed by non-host defences [11–13]. Indeed, plant defences are thought to be the main drivers of host-plant associations in herbivorous insects [1,14]. Given the frequent occurrence of seemingly defended floral rewards, it seems possible that host-plant associations of pollinators could be similarly constrained by their ability to tolerate plant defences.

Dietary specialization among the world's approximately 20 000 bee species is highly variable, and bee larvae differ greatly in their ability to develop to maturity on different pollen hosts, suggesting that as-yet-unknown factors restrict pollen (but not nectar) use in these pollinators [15–20]. In a few instances, pollen defence compounds have been found to harm or even kill bees that are not specialists on the plant taxa that produce these defences [7,21,22]. Moreover, the patterns of host-plant use by bees, such as widespread specialization and phylogenetic conservatism (the tendency for related species to use similar hosts), show striking similarities to those of other herbivorous insects [17,23–25], indicating that host use could be influenced by similar plant characteristics (e.g. plant defences).

Bees’ use of specific host-plant taxa for pollen can also influence their interactions with parasites [26–29]. Some bee parasites, including gut and brood parasites, seem to be harmed by some pollens, but in most cases, we do not know what the harmful properties are; they may or may not be the same ones that influence bee host-plant use. For example, among solitary mason bees, Asteraceae pollen specialists experience much lower rates of brood parasitism by *Sapyga* wasps than do generalists and Fabaceae specialists [26]. Similarly, infection of bee brood by the fungal pathogen *Ascosphaera* is considerably less frequent in the nests of *Ranunculus* specialists than in nests of other, related bee species [30]. In herbivorous insects, plant defences are sometimes co-opted for defence against enemies [12,31]. For example, numerous species of butterflies and beetles gain protection against predators by sequestering secondary metabolites from their host plants [32,33]. Pollen defences could similarly protect specialist bees against parasites [26]—likely a more salient threat than predators, given that most female bees are already well defended against the latter by their stingers—thus potentially mediating bee–parasite interactions.

Here we first ask whether solitary bees parallel other insect herbivores in being tolerant of the defences of their hosts and intolerant of non-host defences. This pattern underlies the important role of plant defences in restricting host-plant use among herbivorous insects [11,12]. From this pattern we can deduce that (1) insect herbivores are typically adapted to the defences of their hosts, and (2) there is a fitness cost to exploiting novel hosts. Finding a similar pattern in solitary bees would suggest that their host use is similarly restricted by plant defences. To test this, we compare the ability of multiple solitary bee species to tolerate pollen defences from their host plants relative to those of co-occurring non-host plants exploited by other, closely related bee species. Specifically, we test the effect of a putative mechanical defence from Asteraceae pollen and a chemical defence from *Lupinus* (Fabaceae) pollen on larval development and survival in nine bee species of the tribe Osmiini, including generalists, specialists on Asteraceae pollen, and a specialist on Fabaceae pollen. We also assess the effects of these putative defences on a pollen-feeding wasp that is the primary brood parasite of several Osmiini species, as pollen defences could protect bees adapted to tolerate such defences against parasites.

## 2. Methodology

### (a) Study species

The tribe Osmiini (Hymenoptera: Megachilidae) includes many pollen specialists and generalists [34,35]. As in most bees, dietary specialization in Osmiini occurs only for pollen, not for nectar, and is typically at the level of plant genera, tribes or families (not individual plant species). Many species of Osmiini nest in abandoned tunnels made by other insects in dead or damaged trees. Osmiini nests consist of a series of individual brood cells, each containing a single egg laid on a pollen-and-nectar provision. The brood cells are produced sequentially along the length of the nest tunnels and are separated by walls made of mud and/or plant material [36]. In osmiine bees, mothers provide all the larval food before the larvae hatch; thus, mothers cannot adjust the content of pollen provisions in response to larval health.

We studied nine Osmiini species, all of which readily nest in artificial nesting cavities (‘trap-nests’; see §2b ‘Nest collection’ below), facilitating nest collection and experimentation. This included five generalist species (*Hoplitis fulgida* (Cresson)*, Osmia lignaria* Say, *Osmia pusilla* Cresson*, Osmia tersula* Cockerell, *Osmia tristella* Cockerell), three Asteraceae specialists (*Osmia coloradensis* Cresson, *Osmia montana* Cresson, *Osmia subaustralis* Cockerell) and one Fabaceae specialist (*Osmia iridis* Cockerell & Titus). The brood cells of four of these species (*O. lignaria*, *O. tersula*, *O. tristella* and *O. iridis*) were frequently parasitized by the brood parasite *Sapyga* sp. (Hymenoptera: Sapygidae; misidentified as *Sapyga pumila* in previous publications), larvae of which kill the host egg before consuming its food provision [26]. This brood parasite was therefore also included in our experiments. Bee diets were characterized by examining the pollen content of hundreds of nests over 10 years (electronic supplementary material, figure S1; see [26] for details) and consulting relevant literature [34,35,37–39]. We considered a species to be a pollen specialist (i.e. oligolectic) when more than 95% of the pollen provision samples examined were dominated by a single plant family. Voucher specimens will be deposited at the Canadian National Collection of Insects, Arachnids and Nematodes (https://www.cnc.agr.gc.ca/taxonomy/TaxonMain.php).

### (b) Nest collection

We collected Osmiini nests using artificial nesting structures that consist of wooden blocks containing holes of three different diameters (to attract different species of bees), affixed to dead or dying trees to mimic the natural cavities in which Osmiini nest (see [40] for details). We lined the holes of the nesting structures with blind-ended paper straws that could be removed to collect bee nests without damaging them. We installed between 6 and 12 artificial nesting structures at each of 12 sites near the Rocky Mountain Biological Laboratory in Colorado, USA (see electronic supplementary material, table S1 for site locations). Bee nests were collected over the summers of 2021 and 2022. Artificial nesting structures were examined approximately twice per week from the middle of June to the beginning of August and newly completed nests were brought to the laboratory for manipulation.

### (c) Pollen defence manipulations

**Figure 1.**
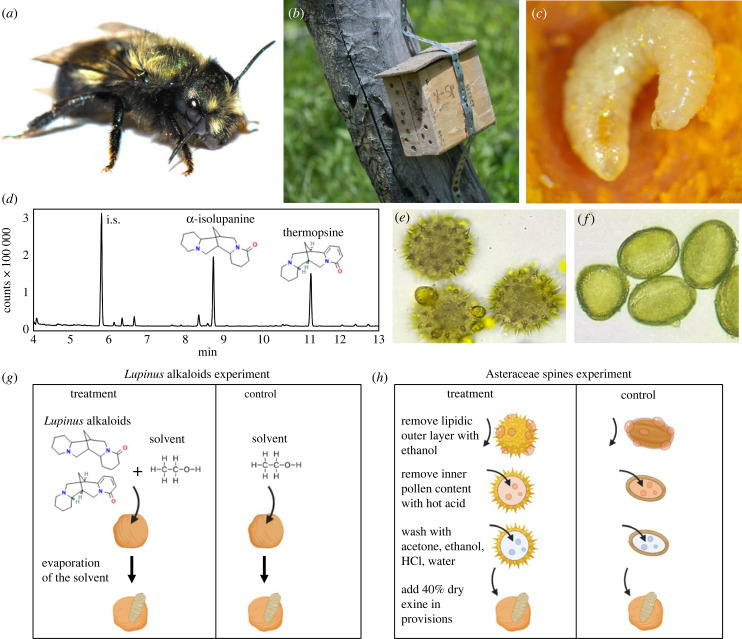
Types of pollen defences and experimental design for the larval provision manipulation experiment. (*a*) *Osmia lignaria*, a generalist species of Osmiini. (*b*) Artificial nesting structure containing holes lined with paper straws used to collect Osmiini nests. (*c*) Larva of *O. montana* on its provision. Larval development and survival were recorded at least every other day. (*d*) Gas chromatography–flame ionization detector (GC-FID) chromatogram showing the pollen alkaloid profile of a *Lupinus argenteus* individual (a common host of generalist Osmiini) from our study site showing peaks for α-isolupanine and thermopsine (i.s. = internal caffeine standard), two of the most common quinolizidine alkaloids in *L. argenteus* pollen. α-Isolupanine and thermopsine were used to manipulate the presence of *Lupinus* pollen alkaloids in Osmiini provisions. (*e*) Asteraceae pollen with visible spines, added to provisions for the Asteraceae pollen spines experiment (following treatment shown in (*h*)). (*f*) Non-spiny pollen used as a control in the Asteraceae pollen spines experiment. (*g*) Methodology for manipulating the provisions in the *Lupinus* alkaloids experiment. (*h*) Methodology for manipulating the provisions in the Asteraceae pollen spines experiment. Photos and illustrations by Sébastien Rivest.

To test the effect of pollen defences on bee larval survival and development, we selected two types of pollen defences from plants that are (1) abundant at our study site, and (2) exploited by some of the investigated Osmiini species but avoided by others. Specifically, we tested the effect of Asteraceae pollen spines and *Lupinus* pollen alkaloids. Asteraceae pollen, like that of a few other plant families, is characterized by conspicuous spines on its surface (figure 1*e*). The exact role of pollen spines in pollination remains unclear, but one study provided evidence that they can deter generalist pollinators [41]. Among our study species, Asteraceae pollen is exploited by Asteraceae specialists, but is rarely observed in the provisions of generalists and Fabaceae specialists (electronic supplementary material, figure S1; see also [16,25]).

The plant genus *Lupinus* (Fabaceae) contains toxic quinolizidine alkaloids in most of its tissues, including its pollen [21,42,43]. Based on pollen sampling from four populations by Heiling *et al*. [43], the population average concentration of quinolizidine alkaloids in *L. argenteus* pollen ranges from 1.51 to 4.03 mg g^−1^ (overall population average = 2.81 mg g^−1^). The main quinolizidine alkaloids in *L. argenteus* pollen near our study site are thermopsine, anagyrine and α-isolupanine ([43]; figure 1*d*). *Lupinus* pollen is common in the provisions of our investigated Osmiini generalists, ranging from being a minor contributor to being the principal pollen, but it was not observed in the provisions of the Asteraceae and Fabaceae specialists (electronic supplementary material, figures S1 and S2). The Fabaceae specialist (*O. iridis*) appears to exclusively use pollen from the tribe Fabeae (*Lathyrus* and *Vicia* spp. in our study area), despite the similarity of the latter in flower size and morphology to *Lupinus*. It is not clear why *O. iridis* does not exploit *Lupinus*, but pollen chemistry could play a role given that, unlike *Lupinus*, plants from the tribe Fabeae do not produce quinolizidine alkaloids [44].

We separated the experiment into two sub-experiments: one for the Asteraceae pollen spines and one for the *Lupinus* alkaloids. All provisions within a nest (i.e. contained within the same paper straw) were assigned to the same sub-experiment. In both sub-experiments, provisions were assigned alternately to the control and defence-addition treatments. Prior to weighing and manipulating the provisions, eggs (or young larvae; see below) were separated from their provisions using spatulas and stored in Petri dishes with moist cotton balls to maintain high humidity. After manipulation of provisions, the eggs were placed back on their own provisions, usually within 1.5 h of being separated from them. Eggs were used when possible, but young larvae (within 10 days of the estimated date on which the egg was laid; in our study area, eggs hatch approximately one week after being laid) were used as well: overall, 33% of bees used as experimental subjects were transferred as eggs and 66% as young larvae. This proportion varied between species but was similar within experiment and species. Brood cells parasitized by *Sapyga* sp., in which the *Sapyga* larva had replaced the host, were also manipulated following the same methodology as for Osmiini.

To manipulate the presence of Asteraceae pollen spines in Osmiini provisions, we added to the provisions empty pollen ‘shells’ of either Asteraceae pollen or, for our control, a mix of non-spinous pollen grains, both subjected to the same procedure. This manipulation allowed us to remove potential confounding effects of pollen nutrients and chemical defences on bee larval development. Honeybee-collected pollen was used in both treatments (Asteraceae pollen was purchased from Changge Huading Wax Industry Co., Henan Province, China; non-spinous pollen was purchased from Alovitox, California, USA). We visually confirmed (using microscope slides stained with basic fuchsine) that the pollen used for the Asteraceae spines treatment contained more than 95% Asteraceae pollen, while the pollen used for the non-spinous treatment contained less than 5% spinous pollen grains. We first removed sugar from the honeybee-collected pollen by washing it in deionized water (following [45]). We then washed the pollen grains in warm ethanol twice to remove the lipidic external layer of the pollen grains (i.e. pollenkitt). We finally removed the inner content of the pollen grains using hot acid reflux with phosphoric acid for 5 h followed by a series of 17 wash steps with solvents (ethanol and acetone), hydrochloric acid, and water (following [46]). We added empty pollen shells (exine) to the provisions at a concentration of 5% by mass, corresponding to approximately 40% by volume (empty exine is considerably lighter than unmanipulated pollen grains). We verified that the empty pollen shells of both treatments occupied approximately the same volume by analysing microscope slides of the pollen content of the manipulated provisions stained with basic fuchsine (using the same method as for the determination of the pollen content of the provisions). The percentage volume of empty pollen shells in the provisions was measured using the image-processing software ImageJ [47]. Because Osmiini pollen provisions are made of a mix of pollen and nectar (in proportions that vary among species, but with more pollen than nectar; [48,49]), we added sterile sugar water (30% sugar, using table sugar) to the provisions to compensate for the volume of empty pollen shells added to the provisions. Sterile sugar water was added in a concentration of 0.15 µl mg^−1^ of the initial provision (before adding the empty shells). This allowed us to obtain a consistency comparable to that of natural provisions. The provisions were mixed with the added pollen shells and sugar water to obtain a homogeneous texture.

To manipulate the presence of *Lupinus* chemical defence we spiked the provisions with a mix of thermopsine (from AOBIOUS, Massachusetts, USA) and α-isolupanine (from ChemFaces, China) in a 5 : 1 ratio and a concentration of 2 mg g^−1^ of provision. (Anagyrine, another of the most commons alkaloids in *L. argenteus* pollen, is a stereoisomer of thermopsine.) This concentration was within the range of quinolizidine alkaloid content in the pollen of *L. argenteus* (see above). Because Osmiini provisions are mainly composed of pollen [48,49], and because *Lupinus* pollen was sometimes the main pollen component in unmanipulated provisions of the generalist species, it seems likely that generalist Osmiini are frequently exposed to the concentration of *Lupinus* alkaloids used in this study. The alkaloids were dissolved in ethanol and the solution mixed with the provisions to allow the alkaloids to be homogeneously distributed. We used a higher proportion of thermopsine than α-isolupanine (rather than mimicking the exact ratio observed in pollen) because the latter had a lower solubility in ethanol. We added ethanol (without alkaloids) to the control provisions. In both treatments, ethanol was allowed to evaporate from the provisions for 1 h before eggs were reintroduced.

Following manipulations, the eggs with their provisions were stored in individual wells (approx. 2 × 1 cm) in wood blocks. A glass or plastic coverslip was taped on the top of each well to allow observation of larval survival and development. The wood blocks containing the eggs or larvae were kept in a growth chamber on a 10–26°C ramping diurnal cycle (a temperature range selected to roughly match daily maximum and minimum values recorded in midsummer by HOBO loggers installed near our study sites; see [50]) and assessed every other day to monitor the bees' survival and developmental stage. Survival was assessed until larvae started spinning their cocoons (from mid-June to mid-October), at which point they had reached the final (fifth) larval instar and had usually stopped feeding. In total, we monitored 413 larvae: 281 for the Asteraceae spine experiment (control = 142, treatment = 139) and 187 for the *Lupinus* alkaloids experiment (control = 93, treatment = 94) (see electronic supplementary material, table S2 for the sample size of each bee species).

### (d) Phylogenetic reconstruction

To be able to account for phylogenetic relatedness in our statistical analyses, we reconstructed a phylogeny for our investigated Osmiini species using the concatenated and aligned matrix from a previously published *Osmia* phylogeny [51], which was reconstructed using DNA sequence data from three nuclear genes (*elongation factor 1-α*, *LW-rhodopsin* and *CAD*) and the mitochondrial *COI* gene. The phylogeny of Rightmyer *et al*. [51] included most of our *Osmia* species, but it did not include *Hoplitis fulgida* or *O. tersula*. However, it did include close relatives of each of these species, so we were able to use the sequence data from those close relatives as proxies: for *H. fulgida*, which was the only *Hoplitis* species in our study, we used sequence data from the closely related *Hoplitis albifrons* (*H. albifrons* and *H. fulgida* belong to the same subgenus); for *O. tersula*, we used data from *Osmia inermis*, which was identified as the nearest neighbour of *O. tersula* by Sheffield *et al*. [52] (both species belong to the same subgenus)—we could not use the Sheffield *et al*. [52] phylogeny for our analyses because it did not include most of our study species. Our results were robust to the removal of *O. tersula* or *H. fulgida*, suggesting that potential errors in branch lengths for these species did not affect our results. We inferred a phylogeny for our nine Osmiini species from the Rightmyer *et al*. dataset under the maximum-likelihood (ML) method on RAxML v.8 [53] and calculated bootstrap proportions via 1000 replicates. The estimated phylogeny was congruent with the tree of Rightmyer *et al*. [51] and had high branch support (greater than 95%). We did not pursue a Bayesian estimation of the phylogeny since the same dataset was found by Rightmyer *et al*. [51] to provide robust estimations under both ML and Bayesian methods. We determined the phylogenetic correlation among Osmiini species using the vcv.phylo function from the R package APE [54].

### (e) Statistical analysis

We conducted all analyses in R v. 4.1.1. [55]. We used Bayesian Cox-proportional hazard models to test the effect of pollen chemical and mechanical defences on bee larval mortality rate. Using Bayesian models allowed us to test the effect of the pollen defences on each bee species simultaneously without introducing problems of multiple testing, while also controlling for phylogenetic relatedness (see below). To compare the effect of the pollen defences among bee species, we first tested the effect of the two pollen defences (in separate models) on all bee species, irrespective of their pollen host. Pollen defence treatment, year and transfer stage (whether the monitored larva was introduced to its provision at the larval or egg stage) were included as fixed effects. Hazard models yield hazard ratios as estimates of treatment effects; hazard ratios greater than 1 indicate greater mortality in the treatment relative to the control group, i.e. a detrimental effect of the defence. As random effects, we included the species identities with their phylogenetic covariance with random intercepts and random slopes (the random slopes allow the effect of the pollen defence treatment to vary between species), as well as the nest's source location (nest, nested within nesting block, nested within site) and the date of transfer with random intercepts. To test if pollen-host use mediates the ability of bees to tolerate pollen defences, we used another set of models that compared the effect of the pollen defences between pollen-host use types (Asteraceae specialists, Fabaceae specialists and generalists). We included the pollen defence treatment (control or pollen defence), the pollen-host use of the bee, an interaction term between those two variables, and year and transfer stage as fixed effects. The interaction between treatment and pollenhost use allowed us to test if bees' abilities to tolerate pollen defences depend on their pollen-host use. An effect was considered significant when the credible interval did not overlap zero. We included the species identities with their phylogenetic covariance, the date of transfer, and the nest location (as above) as random effects. Transfer stage was not a significant predictor in any model and is not discussed further.

The models were run using Hamiltonian Monte Carlo (HMC) sampling, with four chains of 10 000 iterations, of which the first 2500 were discarded as burn-in. We used normal priors (mean = 0, s.d. = 5) for the fixed effects and Student-*t* priors (location = 0, d.f. = 3, scale = 2.5) for the variance of the random intercepts and slopes. These values were chosen to be weakly informative (i.e. slightly regularizing the model without providing information about the expected outcome; see [56]). The thinning intervals were set to 8 to reduce autocorrelation in the Markov chains. Convergence of chains for all parameters was verified both visually with trace plots and with the Gelman–Rubin convergence statistic [57]. The models were run using the brm function from the brms package in R [58].

## 3. Results and discussion

### (a) Pollen defences increase mortality in Osmiini

**Figure 2.**
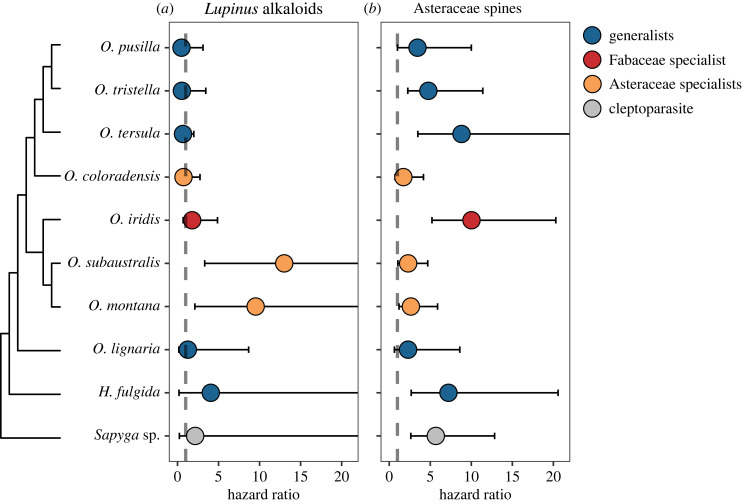
Ratio in mortality rate between larvae in the pollen defence treatment and those in the control (i.e. hazard ratio) for the *Lupinus* alkaloids (*a*) and Asteraceae pollen spines (*b*) experiments. Hazard ratios are shown for nine Osmiini species as well as *Sapyga* sp., a cleptoparasite of *Osmia*. The mean predicted values and the 95% Bayesian credible interval are presented. The credible interval extends beyond the limits of the figure for *Osmia subaustralis*, *Osmia montana*, *Osmia fulgida* and *Sapyga* sp. in (*a*): maximum values = 73.9, 72.0, 210.6, and 24.1, respectively, and for *Osmia tersula* in (*b*): maximum value = 26.5.

We first tested whether naturally occurring concentrations of pollen chemical defence from *Lupinus* and mechanical defence from Asteraceae are detrimental to larval survival in Osmiini. We found that both types of defences are harmful to some Osmiini species, with each defence affecting a different subset of the investigated species (figure 2; electronic supplementary material, table S2). Specifically, *Lupinus* alkaloids strongly increased the mortality rate of two Asteraceae-specialist Osmiini (*O. subaustralis* and *O. montana*) (figure 2*a*) but had no noticeable impact on the mortality of generalists (*H. fulgida*, *O. lignaria*, *O. pusilla*, *O. tersula* and *O. tristella*) or the Fabaceae specialist (*O. iridis*). Conversely, Asteraceae spines were detrimental to the survival of the Fabaceae specialist and some generalists (*O. tristella*, *O. tersula* and *H. fulgida*), while other generalists (*O. pusilla* and *O. lignaria*) and the Asteraceae specialists (*O. coloradensis*, *O. montana* and *O. subaustralis*) exhibited little to no impact from this putative defence (figure 2*b*). In species strongly harmed by pollen defences, the hazard ratio—the ratio in mortality rate between the treatment and control larvae—typically ranged from 4 to more than 10 (figure 2). These multiple-fold increases in mortality rate show that pollen defences could have substantial impacts on the fitness of Osmiini bees. Moreover, similar mortality rates are common in solitary bee larvae reared on non-host pollen, providing support for the hypothesis that pollen defences contribute to the frequently observed unsuitability of non-host pollen for bee larval development [16,18,20,48,59].

### (b) Osmiini better tolerate the defences of their hosts relative to non-host defences

**Figure 3.**
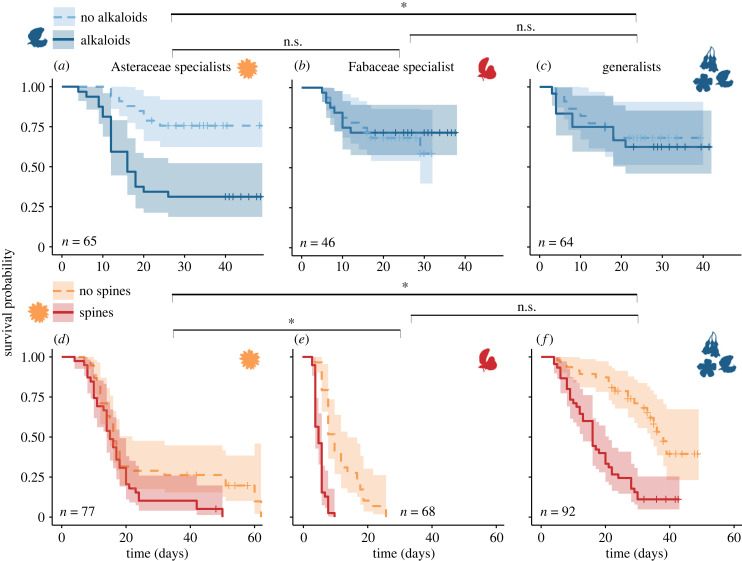
Survival curves of Osmiini bees exposed or not to *Lupinus* alkaloids (*a–c*) or Asteraceae pollen spines (*d–f*) as a function of their host-use types: Asteraceae specialists (*a, d*), Fabaceae specialist (*b, e*), and generalists (*c, f*). Significant differences (95% Bayesian credible interval does not overlap zero) in tolerance of a given defence between host-use types are represented by asterisks.

Given that pollen defences can affect survival in Osmiini, we then compared the ability of these bees to tolerate host relative to non-host defences. We found that the ability of solitary bee species to tolerate different pollen defences is mediated by their host-plant use, with bees being more tolerant of the pollen defences of their hosts relative to defences from non-host plants exploited by other Osmiini species. Specifically, generalist Osmiini, which often exploit *Lupinus* as a pollen host, better tolerated *Lupinus* pollen alkaloids than did Asteraceae specialists (Bayesian credible interval of the difference in hazard ratio between host-use types: 95% CI = 0.91 to 3.53), although the Fabaceae specialist (which also does not use *Lupinus* pollen) was no more affected than generalists by this defence (CI = −0.68 to 2.15) (figure 3*a–c*). Similarly, Asteraceae pollen spines were more harmful to Osmiini species that do not, or rarely, exploit Asteraceae pollen (Fabaceae specialist and generalists) relative to Asteraceae specialists (CI = 1.19 to 2.92 for Fabaceae versus Asteraceae specialists, and 0.60 to 2.23 for generalists versus Asteraceae specialists) (figure 3*d–f*).

Overall, the fact that defences from pollen hosts readily exploited by some bee species are harmful to other species suggests that, like other insect herbivores, solitary bees are physiologically or morphologically adapted to the defences of their hosts [6,18,60]. In turn, by showing that these pollinators often lack tolerance of non-host pollen defences, our results provide strong evidence that such defences constitute a constraint in solitary bee pollen-host use. As in other insect herbivores, the exploitation of plant-hosts by bees might often require overcoming certain detrimental effects of pollen chemistry and morphology, perhaps by evolving physiological adaptions to pollen properties or via behavioural mechanisms that reduce exposure to them (e.g. pollen mixing; [19]).

Notwithstanding these general patterns, some Osmiini species were highly tolerant of non-host pollen defences. For example, while the two closely related Asteraceae specialists, *O. montana* and *O. subaustralis*, were detrimentally affected by *Lupinus* alkaloids, the more phylogenetically distant *O. coloradensis* tolerated these compounds (figure 2*a*). Similarly, the Fabaceae specialist *O. iridis* was not affected by alkaloids from *Lupinus*, a genus closely related to the floral hosts of *O. iridis*. The lack of effect of lupine alkaloids on *O. iridis* suggests that these bees avoid lupine flowers for other reasons, such as other chemical constituents of the pollen or the fact that lupine flowers do not produce nectar. The capacity of some Osminii species to tolerate non-host defences is not unexpected, given that similar tolerance is observed in other insect herbivores as well [61,62]. In bees and other insect herbivores, the ability to use non-host plants is thought to occur when species have not yet lost adaptations to ancestral hosts, despite transitions to new hosts having occurred. In bees, evidence for this mechanism of non-host tolerance comes from the fact that diet expansion often occurs through the adoption of pollen hosts exploited by related species, suggesting that phylogenetically conserved adaptations are frequent [17].

Irrespective of treatment, larval mortality was higher in the Asteraceae spines sub-experiment than in the *Lupinus*-alkaloids sub-experiment (figure 2). This difference likely occurred because in order to isolate the mechanical properties of pollen grains in the Asteraceae spines experiment, empty pollen ‘shells’, rather than nutrient-filled pollen grains, were added to the provisions (at approx. 40% v/v), which reduces the nutrient concentration in larval provisions. This lower nutrient concentration is, however, unlikely to be responsible for the difference we observed between treatments, since the proportion of empty exine was similar between spiny and non-spiny (control) pollen treatments (5% m/m in both treatments, corresponding to v/v of 39.9 ± 5.0% for non-spiny and 36.4 ± 6.1% for spiny exine; see Methodology). In this experiment, Asteraceae-specialist Osmiini represented a good control to assess whether we effectively isolated the relevant mechanical properties of pollen grains because these species are expected to tolerate pollen spines relatively well. We found little to no detrimental impact of pollen spines relative to non-spinous pollen shells in these latter species (CI = −0.03 to 1.00, figures 2*b*, 3*d–f*), reinforcing our conclusion that pollen mechanical properties are responsible for the strong detrimental effect of spinous pollen exine on generalists and Fabaceae specialists.

### (c) Pollen defences protect specialist bees against a cleptoparasite

In addition to Osmiini, we investigated the impact of pollen defences on a common cleptoparasite of *Osmia* spp., *Sapyga* sp. In our study area, this *Sapyga* species frequently parasitizes Fabaceae specialists and generalists but does not occur in the nests of Asteraceae specialists [26]. Moreover, a previous egg-transfer experiment has shown that the mortality rate of *Sapyga* wasps is higher on Asteraceae provisions than on the provisions of Fabaceae specialists and generalists [26]. Our results show that Asteraceae pollen spines increase the mortality rate of *Sapyga* wasps, suggesting that this pollen defence is responsible for the protective function of Asteraceae pollen against cleptoparasites (figure 2*b*). By contrast, we did not detect an effect of alkaloids from *Lupinus*, a pollen type frequently present in the provisions of *Sapyga* hosts, on *Sapyga* mortality (figure 2*a*). This finding provides evidence that specialization on putatively defended pollen hosts can confer fitness advantages in bees via its protective effect against antagonists. Wynns [30] found evidence for a similar protective function of the protoanemonine from *Ranunculus* (Ranunculaceae) pollen against parasitic fungi in bees specialized on this pollen, while Figueroa *et al*. [29] found that Asteraceae spines can protect *Bombus impatiens* against a common gut pathogen. Hence, parasites and pathogens could impose selection favouring specialization on defended pollen in bees, a pattern akin to the co-opting of defences by other kinds of specialized herbivores [12,31].

### (d) The role of pollen defences in the ecology and evolution of bees

Despite bees and other pollen consumers seemingly facing an array of defences, little is known about how these putatively antagonistic traits affect the ecology and evolution of pollinators. Here we demonstrate a striking parallel between solitary bees and other phytophagous insects: like other phytophages, bees tolerate the defences of a restricted subset of potential hosts while often being harmed by the defences of non-host plants. Moreover, in bees and in other herbivorous insects, specialization on defended hosts can confer protection against antagonists. These findings, in combination with previous studies showing lethal or sublethal effects of pollen secondary metabolites on bees [7,21,22], suggest that pollen defences might be an important driver of pollen-host use in bees.

Multiple lines of evidence indicate that bees are highly constrained in their pollen diet. First, the fact that bees often specialize on specific hosts for pollen but rarely for nectar suggests that pollen requires more taxon-specific adaptations to acquire and metabolize [15], a pattern that coincides with the typically higher concentration of secondary metabolites in pollen than in nectar [5,6]. Perhaps more revealing is the remarkable similarity between the evolutionary patterns of host use in bees and other insect herbivores, the latter being mostly attributed to plant defences [1,14,17,23–25,63,64]. This similarity is not only superficially suggestive; the evolutionary patterns in question—phylogenetic conservatism, widespread specialization, and the integration of new hosts exploited by related species—usually manifest when host use is constrained by host characteristics of some kind [17,24,63]. For example, phylogenetically conserved host associations should occur when host use is not labile, leading to restricted ability to integrate new hosts or switch hosts over evolutionary timescales [6,63,64]. Pollen defences should at least partially contribute to these patterns since they appear to restrict solitary bees' ability to exploit new pollen hosts. In other words, the often drastic increase in mortality associated with feeding on non-host pollen that we observed across Osmiini species should limit the ability of solitary bees to adopt novel hosts whose defences they do not tolerate.

As with other insect herbivores, plant defences likely interact with other plant attributes in determining host suitability for bees. For example, pollen nutrient content and nutrient ratios have been shown to potentially mediate the host preferences of multiple bee species that might have different nutritional needs [20,65–69]. The mechanical fit between bees and their floral hosts can also restrict host use (although this phenomenon has been studied mostly in the context of nectar collection) ([70–73], but see [74]), as does the availability of different hosts in space and time [17,75,76]. In our study system, the fact that *O. iridis* does not collect pollen from *Lupinus* despite tolerating its chemical defences and exploiting related plants supports the role of multiple factors in determining bee host use.

Our results corroborate the hypothesis [17,18] that the striking similarity between the evolutionary patterns of host use in bees and other herbivorous insects is due to their diet being restricted by the same host characteristics: plant defences—both chemical and morphological. As we demonstrate in this study, pollen defences are not just a botanical curiosity, but, rather, likely play an integral role in the ecology and evolution of plants' main pollinators, bees. Considering pollination through this antagonistic perspective—by investigating the causes and consequences of pollen defences—promises to improve our understanding of the intricate interactions between plants and their pollinators.

## Data Availability

Data and code used for this manuscript can be accessed via the Dryad repository: https://doi.org/10.5061/dryad.h44j0zpsx [77]. Supplementary material is available online [78].
