# Correction to: ‘Pollen chemical and mechanical defences restrict host-plant use by bees’ (2024), by Rivest *et al*.

**DOI:** 10.1098/rspb.2024.0578

**Published:** 2024-03-25

**Authors:** Sébastien Rivest, Madhupreeta Muralidhar, Jessica R. K. Forrest

**Affiliations:** Department of Science, University of Ottawa, Ottawa, Ontario Canada, K1N 6N5

**Keywords:** pollen, bees, secondary metabolites, defence compounds, pollinators, flowers

*Proc. R. Soc. B* **291**, 20232298 (Published online 27 February 2024). (https://doi.org/10.1098/rspb.2023.2298)

While preparing voucher specimens, we became aware that a specimen previously identified as *Osmia tersula* was in fact *O. pikei*. It is possible that other individuals labelled as *O. tersula* in this study were also misidentified; however, this cannot be verified, as the others did not survive to adulthood. *Osmia tersula* and *O. pikei* are closely related species belonging to the same subgenus (*Melanosmia*); both have a generalist pollen diet (for *O. pikei*, see Cripps and Rust [1,2]). Furthermore, as reported in the original article, our results are robust to the exclusion of *O. tersula*’. Therefore, the conclusions of our study are not affected by the identification error.
